# Pharmacokinetics and pharmacodynamics of HTD1801 (berberine ursodeoxycholate, BUDCA) in patients with hyperlipidemia

**DOI:** 10.1186/s12944-020-01406-4

**Published:** 2020-11-12

**Authors:** Adrian M. Di Bisceglie, Gerald F. Watts, Philip Lavin, Meng Yu, Ru Bai, Liping Liu

**Affiliations:** 1 Department of Internal Medicine, Saint Louis University, St. Louis, USA; 2 HighTide Therapeutics, Rockville, MD USA; 3 Department of Cardiology, Royal Perth Hospital, School of Medicine, University of Western Australia, Perth, Australia; 4 Boston Biostatistics Research Foundation, Framingham, MA USA

**Keywords:** Hyperlipidemia, Berberine, Ursodeoxycholic acid, Pharmacokinetics

## Abstract

**Background:**

Reduction in elevated serum cholesterol concentrations is important in the management of individuals at risk of atherosclerotic cardiovascular disease (ASCVD), such as myocardial infarction and thrombotic stroke. Although HMGCoA reductase inhibitors (“statins”) are frequently used for this purpose, a significant proportion of patients remain at increased residual risk of ASCVD as they do not adequately address some of the associated co-morbidities such as diabetes and fatty liver disease.

**Methods:**

A double-blind, randomized, placebo-controlled, dose ranging study was carried out that compared three doses of berberine ursodeoxycholate (BUDCA) to placebo in a cohort of subjects with a history of hypercholesterolemia and serum LDL cholesterol levels above 2.59 mmol/L (> 99.9 mg/dL). BUDCA was administered in two divided doses each day for 28 days. The primary endpoints of the study were safety and tolerability of this new compound, as well as its effect in lowering serum lipid and lipoprotein concentrations.

**Results:**

A total of 50 subjects were enrolled into three dose cohorts in this study. BUDCA was generally well tolerated, even at doses of 2000 mg per day (the highest dose group); there were no significant adverse effects reported and this highest dose was associated with significant reductions in LDL cholesterol. By day 28 and with the highest dose of BUDCA, there were significant reductions in the serum concentrations of total cholesterol by 8.2% (*P* = 0.0004) and LDL cholesterol by 10.4% (*P* = 0.0006), but no significant changes in triglyceride and HDL cholesterol concentrations.

**Conclusions:**

BUDCA is a new single molecular entity that has a significant but modest effect in safely lowering serum LDL-cholesterol concentrations in individuals with a history of hypercholesterolemia. It has a potential use for treating hypercholesterolemia in individuals who cannot take statins, and possibly as adjunctive to other agents, such as ezetimibe or bempedoic acid.

**Trial registration:**

The study was registered on Clinicaltrials.gov (NCT03381287).

## Introduction

Dyslipidemia is a major risk factor for atherosclerotic cardiovascular disease (ASCVD) and encompasses elevation in the plasma concentrations of low density lipoproteins and/or triglyceride-rich lipoproteins [1]. Reduction in plasma LDL-cholesterol concentrations is the primary treatment target of all contemporary major guidelines for patients with and without ASCVD [2, 3]. These targets may be achieved with diet, exercise and use of moderate or high potency statins. Many patients, however, remain at high residual risk of ASCVD and this may be related to associated co-morbidities, such as diabetes and fatty liver disease [4, 5], or because they are intolerant to statins [6–8]. Non-statin therapies, such as ezetimibe, bempedoic acid, PCSK9 inhibitors and nutraceuticials, are therefore required to address this gap in treatment [9, 10]. Cholesterol lowering agents that have beneficial effects of insulin resistance, diabetes and non-alcoholic fatty liver are also highly desirable [4, 5].

Berberine ursodeoxycholate (BUDCA) is a new molecular entity, created as an ionic salt formed between berberine (BBR) and ursodeoxycholic acid (UDCA). BBR is a plant-extracted compound with anti-oxidant and anti-microbial properties [11–13], available without a prescription and widely used for its benefits in diabetes and cardiovascular disease. There is good evidence that BBR has beneficial effects in lowering serum cholesterol levels [14, 15], but the BBR products available over the counter are of variable quality and are not considered to be of pharmaceutical grade. Ursodeoxycholic acid (UDCA) is a naturally-occurring bile acid that was originally developed as a therapeutic agent to aid in dissolution of gallstones [16], but has become the standard of care for the chronic cholestatic liver disease primary biliary cholangitis [17, 18] and is also used off-label for a variety of other cholestatic liver diseases [19]. UDCA also has cholesterol-lowering effects [20]. This study was conducted to examine the safety and pharmacokinetic (PK) effects of BUDCA (by separately measuring levels of BBR and UDCA in serum) and responses in serum lipid and lipoprotein concentrations, particularly LDL-C.

## Materials and methods

Adult subjects were recruited and enrolled into this double-blind, randomized, placebo-controlled dose ranging study at one of three research units in Australia between April and December of 2018. The key entry criteria were hypercholesterolemia, with serum LDL > 2.59 mmol/L (> 99.9 mg/dL) and overweight or obesity (BMI between 25 and 40 kg/m^2^). Subjects were otherwise generally healthy, and were excluded if they had a history of cardiac disease, severe, uncontrolled diabetes or other systemic disorders that might limit their participation in the study. Subjects were asked to discontinue the use of lipid-lowering medications at least 28 days prior to their first dose of BUDCA and for the duration of the study.

Subjects underwent an initial assessment and were then randomized to receive ascending doses of BUDCA in three sequential dosing groups: Group I received 500 mg/day of BUDCA or matching placebo in a ratio of 3:1. Group II received 1000 mg/day of BUDCA or placebo, 3:1 and Group III received 2000 mg/day BUDCA or placebo, 3:1. Study medication or placebo was given in two equal doses daily for 28 days. The investigational product BUDCA was administered in the form of film-coated tablets, each tablet containing 250 mg of BUDCA. The placebo was identical in appearance to the active agent. Subjects were instructed to take their tablets after breakfast and after dinner respectively with a glass of water.

**Fig. 1:**
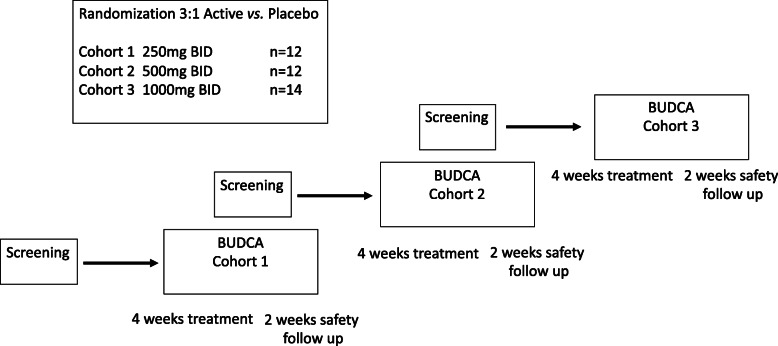
Study overview and randomization scheme

The groups were enrolled sequentially, but follow-up overlapped (the next cohort could begin during the follow-up period of the prior cohort) (Fig. 1). The decision to move to the next dose level was based upon tolerability and safety findings, as assessed by a Safety Monitoring Committee. Individual subjects were admitted overnight for their first and last doses and so as to draw blood samples for pharmacokinetic studies; in between, they were seen every few days. Subjects were asked to arrive in a fasting state (typically overnight). After having vital signs measured and baseline laboratory tests taken, the subject was given a morning meal and then observed while taking their first dose of BUDCA with a glass of water. They stayed overnight to allow sampling for PK studies which were drawn at the following time points − 18 h, − 12, − 6, 0 time (pre-dose), + 0.25 (post-dose), + 0.5, + 1, + 2, + 3, + 4, + 8, + 12, + 24 h. Blood samples for PK studies were collected in dipotassium ethylene diamine tetra-acetic acid (K2EDTA) as the anticoagulant and the assays were done on plasma.

Blood samples for serum biochemistry (including lipid levels) and blood counts were drawn with subjects in a fasting state, at baseline and every 14 days through the study. Samples were shipped in a refrigerated state to a central laboratory (SydPath, Darlinghurst, Australia) where testing was done using routine clinical methodology, as follows: HbA1c was measured by ion-exchange HPLC (Bio-Rad D-100). For samples received by the laboratory prior to September 2nd 2018, the following analytes were measured on the Roche Diagnostics Modular P100 platform: ALT by photometric assay, total cholesterol by Trinder peroxidase assay, LDL-c by photometric assay, HDL-c by homogenous enzymatic colorimetric assay and triglycerides by photometric assay. For samples received by the laboratory after September 2nd 2018, the following analytes were measured on the Beckman Coulter AU 5800 platform: ALT by photometric assay, total cholesterol by cholesterol-oxidase/peroxidase assay, LDL-c by homogenous enzymatic colorimetric assay, HDL-c by immune inhibition and cholesterol oxidase assay and triglycerides by an enzymatic method using opiase/GK/GPO-PAP-4 amino phenazone.

Subjects received dietary counseling with regard to healthy eating habits and use of study medication at several times points in the study. For the pharmacokinetic profile of BUDCA, serum levels of BBR and UDCA were measured after single and multiple doses. PK sampling was done at multiple intervals before and up to 24 h after the first dose of study drug on day 1 and again on day 28.

Concentrations of BBR and UDCA in plasma were determined simultaneously by liquid chromatography tandem-mass spectrometry (LC-MS/MS). The assay was developed by a third party vendor (TetraQ, Mount Waverley, Victoria, Australia) based on pre-clinical in-house assays developed by HighTide. Although assays for both berberine and UDCA have been commonly used, the challenge for this study was to measure both analytes simultaneously in the same sample; hence the need for further methodology development beyond what has previously been done for berberine and UDCA separately, as these two analytes are quite different.

Results were provided to the study sponsor in the form of two written reports, one being a bioanalytical report (i.e. the methodology) and the other being the PK data (data on file). Briefly, 200 μL plasma samples were spiked with 50 μL of the internal standards dissolved in methanol (for berberine BBR-d6; for UDCA, UDCA-d4) and a neutral pH maintained by addition of phosphate buffer (200 mM, pH 7.0). The analyte and standard were then recovered from the matrix by liquid-liquid extraction with methyl *tert* butyl ether (M*t*BE):dichloromethane DCM (65:35). After centrifugation, the supernatant was transferred to a clean tube and evaporated under air at 37 °C. Following reconstitution with 200 μL of 0.1% formic acid in 30% methanol:water, the sample was injected into a Shimadzu Nexera uHPLC system using a Phenomenex Kinetex EVO C18 column for separation. The Multiple Reaction Monitoring (MRM) transition was then monitored on a Sciex 5500 QTrap mass spectrometer coupled with a Shimadzu Prominence μHPLC for separation and detection. The transitions used for berberine, berberine-d6, ursodeoxycholic acid and ursodeoxycholic acid-d4 were 336.1 → 292.0 *m/z*, 342.1 → 292.0 *m/z*, 391.1 → 373.2 *m/z* and 395.2 → 377.2 *m/z* respectively.

Peak area and area ratios were determined electronically by Analyst software version 1.7. Following integration of the analyte and IS peaks, the peak area ratios (analyte/IS) are plotted against nominal analyte concentrations for the standards. The dynamic range of this assay was 0.01–5.00 ng/mL for BBR and 30–6000 ng/mL for UDCA. Pharmacokinetic analyses were also conducted by TetraQ, using Phoenix® WinNonlin® Version 8.1 (Pharsight®, a Certara™ company). Pharmacokinetic parameters were determined using non-compartmental methods (Model 200–202, Extravascular Input) and a linear-trapezoidal approach.

The study was registered on Clinicaltrials.gov (NCT03381287) and the study was conducted in accordance with the International Council for Harmonisation tripartite guideline on the ethical principles of Good Clinical Practice (ICHE6). The study was approved by Independent Ethics Committees (IECs) at each of the participating institutions and all subjects gave written informed consent to participate.

### Statistical analysis

Data from all cohorts were summarized by treatment group in the order: Placebo, BUDCA 500 mg, BUDCA 1000 mg, and BUDCA 2000 mg. Mean changes and percent changes from baseline were computed for lipids, glucose, liver function, hematology, and serum chemistry. Percentage measures involving continuous measures were computed for each patient and then averaged while percentage calculations involving rates used the number of subjects in the relevant population, unless otherwise stated. Pre-planned ANOVA analyses were conducted to perform global comparisons for the separate changes from baseline to Days 14 and 28 across all four treatment groups in order to control Type 1 error for making pre-planned comparisons of each active dose vs. placebo; log transformations were used to normalize the inherent variability in clinical laboratory results; if the ANOVA analyses achieved statistical significance, then two-sided *p*-values were justified to compare each active dose group vs. the pooled placebo controls using unpaired comparisons from ANOVA output. SAS version 9.3 was used for all analyses.

## Results

Fifty subjects were enrolled, including 30 females, equally distributed across the three dosing groups, 38 receiving BUDCA and 12 placebo. The baseline characteristics of enrolled subjects are shown in Table 1. Their mean LDL levels were 3.86 mmol/L (range 2.5 to 5.9). Only three had a history of diabetes or glucose intolerance and Hb1Ac levels were mostly in the normal range.

**Table 1:** Baseline Subject Characteristics and Biochemistry Profile

Characteristic	Placebo	BUDCA Dose		
		500 mg/day	1000 mg/day	2000 mg/day
Number of Subjects	12	12	12	14
Mean age, yrs. (range)	53 (26–63)	48 (27–70)	54 (42–63)	52 (22–70)
Gender (% female)	75%	50%	42%	71%
Race (% white)	92%	100%	100%	100%
Mean weight, kg (range)	88 (68–100)	95 (71–147)	88 (70–121)	84 (63–117)
Mean BMI (kg/m^2^)	30.9 (26–39)	31.1 (27–39)	29.5 (25–35)	30.3 (25–40)
History of diabetes or glucose intolerance	0	0	1	2
History of Hypertension	3	4	2	1
Mean Total Cholesterol (mmol/L)	6.06 (4.6–10.8)	6.09 (4.4–7.4)	5.96 (4.6–7.1)	6.11 (4.5–8.3)
Mean LDL (mmol/L)	3.99 (2.7–5.4)	4.09 (2.8–5.8)	3.57 (2.7–5.1)	3.78 (2.5–5.9)
Mean triglycerides (mmol/L)	1.98 (0.6–6.8)	2.11 (0.6–4.6)	1.65 (0.4–4.4)	1.50 (0.7–3.3)
Mean HbA1c (%)	5.3 (4.9–5.7)	5.4 (4.9–6.0)	5.2 (5.0–5.5)	5.6 (5.1–7.4)
Mean ALT (U/L)	20 (12–56)	26 (7–46)	24 (13–43)	20 (12–42)

**Fig. 2:**
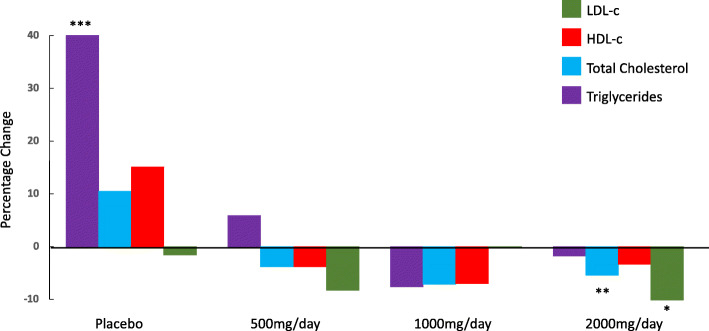
Percentage change in serum lipids at day 28. Marked parameters were statistically significantly different from baseline (*P = 0.0006; ** *P* = 0.0004; *** *P* = 0.0006)

Among the subjects receiving active therapy, serum lipid levels decreased in a dose-dependent fashion (see Table 2 and Fig. 2), with maximum reductions relative to placebo being observed after 14 days of treatment, with little further reduction beyond that. HbA1C and ALT levels did not change significantly during therapy. Significant global differences were observed between study treatment via ANOVA for Triglycerides (Day 28 change from baseline), Total Cholesterol (both Days 14 and change from baseline), LDL-C (both Days 14 and 28 change from baseline), and non-HDL- C (Days 14 and 28 change from baseline). The following pairwise comparisons vs. placebo for the changes from baseline were significant:

- Triglycerides: Day 28 1000 mg vs. placebo (*P* = 0.028) and Day 28 2000 mg vs placebo (*p* = 0.0018)
- Total Cholesterol: Days 14 and 28 2000 mg vs. placebo (*P* = 0.01 and 0.003)
- LDL-C: Day 14 2000 mg vs. placebo (*P* = 0.0093)
- Non HDL-C: Days 14 and 28 2000 mg vs. placebo (*P* = 0.0018 and *P* = 0.002), Day 28 1000 mg vs. placebo (*P* = 0.025) and Day 28 500 mg vs. placebo (*P* = 0.03).

**Table 2:** Change in serum lipids and lipoproteins with berberine ursodeoxycholate therap

Lipid	Time	Placebo	500 mg/day	*P* Value vs. placebo	1000 mg/day	*ANOVA P* Value vs. placebo	2000 mg/day	*ANOVA P* Value vs. placebo
Triglycerides	Baseline*	1.91	2.11	–	1.76	–	1.60	–
	Day 14	1.84	1.89	n.s.	1.73	n.s.	1.42	n.s.
	Day 28	2.58	2.27	n.s.	1.86	0.028	1.54	0.0018
Total Cholesterol	Baseline*	6.23	6.13	–	5.81	–	5.91	–
	Day 14	6.39	5.96	n.s.	5.51	n.s.	5.37	0.01
	Day 28	6.59	5.88	n.s.	5.54	n.s.	5.42	0.003
LDL Cholesterol	Baseline*	3.91	4.01	–	3.68	–	3.85	–
	Day 14	4.05	3.87	n.s.	3.42	n.s.	3.40	0.0093
	Day 28	3.85	3.72	n.s.	3.51	n.s.	3.44	0.091
Non-HDL Cholesterol	Baseline*	4.66	4.85	–	4.48	–	4.62	–
	Day 14	4.90	4.75	n.s.	4.23	n.s.	4.04	0.0018
	Day 28	5.18	4.71	0.03	4.31	0.025	4.14	0.0002
HDL Cholesterol	Baseline*	1.55	1.25	–	1.32	–	1.24	–
	Day 14	1.49	1.22	n.s.	1.29	n.s.	1.33	n.s.
	Day 28	1.42	1.17	n.s.	1.23	n.s.	1.29	n.s.

*Baseline value is calculated from mean of Screening and Baseline visits

Pharmacokinetic analyses showed that the half-life of BBR in serum was about 8 to 10 h and for UDCA approximately 7 h. After repeated dosing, accumulation was about 4-fold for BBR and 2-fold for UDCA. After single oral administration of BUDCA on day 1, peak BBR plasma concentration occurred at a median value of approximately 4.0 h (Tables 3 and 4). A typical half-life ranged from approximately 8 to 10 h and was comparable across doses. Time of the last quantifiable plasma concentration was 24 h across doses. Following multiple oral administration of BUDCA, peak BBR plasma concentrations on day 28 occurred at a median of approximately 3.5 to 4.0 h. Berberine exposure increased approximately 2-fold (C_max_) or 4-fold (AUC_last_) after repeat dosing with BUDCA.

**Table 3:** Summary of pharmacokinetic parameters for berberine after single and multiple doses of BUDCA

	Parameter	250 mg/dose	500 mg/dose	1000 mg/dose
Day 1	AUC_last_ (hr^.^ng/mL)	2.9 (± 1.4)	3.3 (± 2.4)	7.2 (± 3.8)
	C_max_ (ng/mL)	0.4 (± 0.2)	0.4 (± 0.3)	0.9 (± 0.5)
	T_max_ (hr)	3.5 [2.0–8.3]	4.0 [2.0–8.0]	4.0 [3.0–12.0]
	T_1/2_ (hr)	9.0 (± 1.5)	10.6 (± 2.5)	7.8 (± 0.6)
Day 28	AUC_last_ (hr^.^ng/mL)	11.0 (± 4.3)	17.0 (± 9.1)	28.3 (± 21.2)
	C_max_ (ng/mL)	0.68 (± 0.2)	1.5 (± 1.2)	1.8 (1.3)
	T_max (_ng/mL)	4.0 [0–4.0]	4.0 [0.3–12.0]	4.0 [2.0–12.0]
	T_1/2_ (hr)	N.D.

**Table 4:** Summary of pharmacokinetic parameters for UDCA after administration of single and multiple doses of BUDCA

	Parameter	250 mg/dose	500 mg/dose	1000 mg/dose
Day 1	AUC_last_ (hr^.^ng/mL)	3680 (± 1280)	8530 (± 4600)	15,500 (± 5830)
	C_max_ (ng/mL)	923 (± 453)	1730 (± 873)	2900 (± 1520)
	T_max_ (hr)	2.0 [0.5–4.0]	3.0 [1.0–4.1]	4.0 [1.0–8.0]
	T_1/2_ (hr)	2.8 (± 0.9)	6.8 (± 9.9)	5.2 (± 1.6)
Day 28	AUC_last_ (hr^.^ng/mL)	12,000 (± 16,900)	15,100 (± 6780)	2940 (± 10,600)
	C_max_ (ng/mL)	1660 (± 275)	1940 (± 801)	3370 (966)
	T_max (_ng/mL)	3.0 [2.0–12.0]	4.0 [2.0–8.0]	3.5 [0–4.0]
	T_1/2_ (hr)	N.D.	6.7 (± 2.9)	7.5 (± 2.8)

*AUC*_*last*_ area under plasma concentration-time curve to last quantifiable measurement, *C*_*max*_ maximum observed plasma concentration_,_ *T*_*max*_ time to maximum concentration_,_ *T*_*1/2*_ terminal half life

**Fig. 3:**
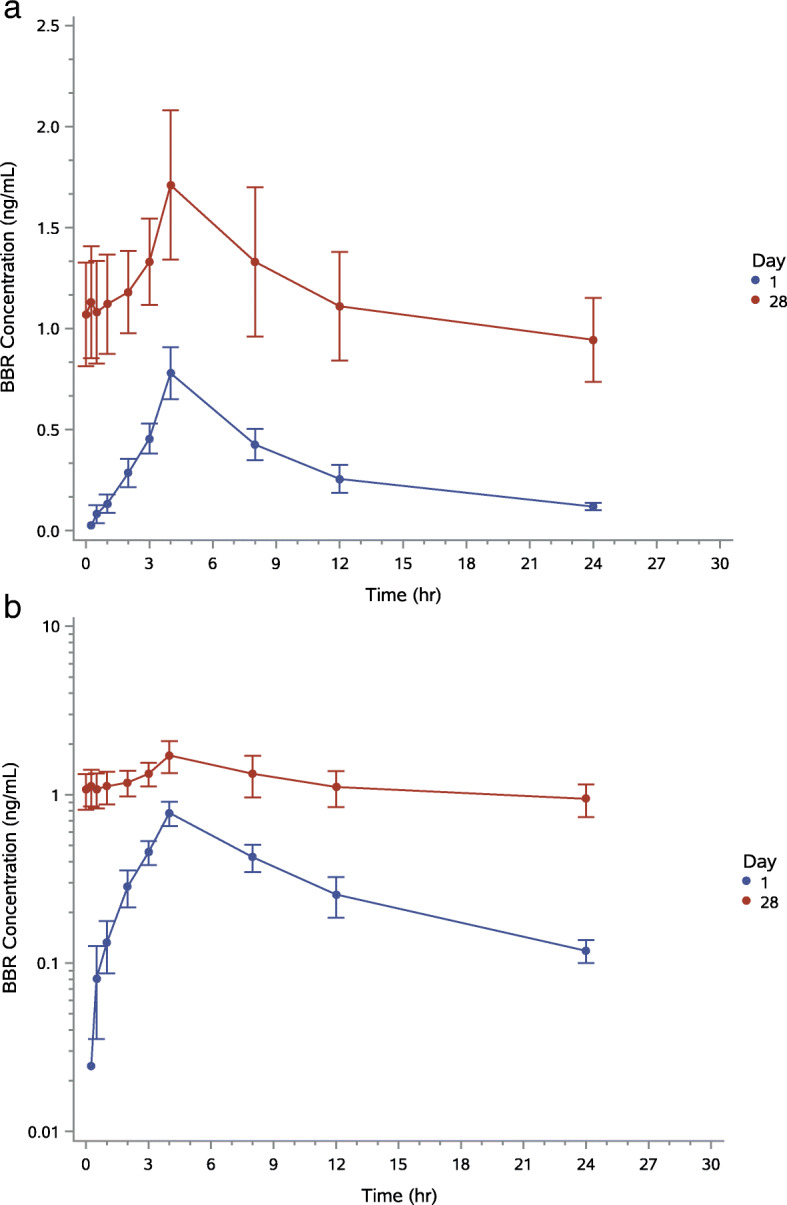
**a** Average (+/−SE) plasma berberine concentration-time profiles (linear plot) after single- (day 1) and multiple-dose administration of HTD1801 in the group receiving the highest dose (1000 mg BID). **b** Average (+/−SE) plasma berberine concentration-time profiles (log-linear plot) after single- (day 1) and multiple-dose administration of HTD1801 in the group receiving the highest dose (1000 mg BID)

**Fig. 4:**
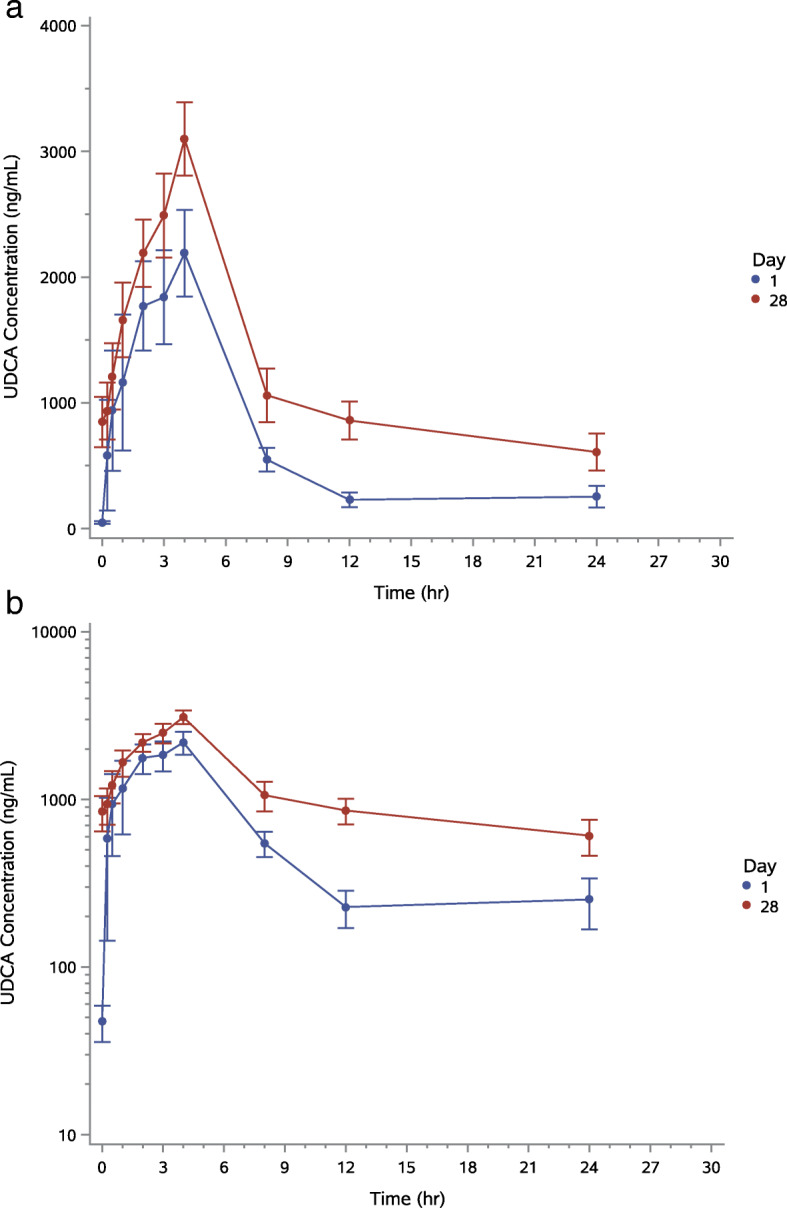
**a** Average (+/−SE) plasma UDCA concentration-time profiles (linear plot) after single- (day 1) and multiple-dose administration of HTD1801 in the group receiving the highest dose (1000 mg BID). **b** Average (+/−SE) plasma UDCA concentration-time profiles (log-linear plot) after single- (day 1) and multiple-dose administration of HTD1801 in the group receiving the highest dose (1000 mg BID)

After single oral administration of BUDCA on Day 1, peak UDCA plasma concentration occurred at a median value of approximately 2.0 h for the 250 mg dose, 3.0 h for the 500 mg dose and 4.0 h for the 1000 mg dose (Table 4). T_max_ values were comparable across doses. The typical half-life of UDCA ranged from approximately 3 to 7 h. Time of the last quantifiable plasma concentration was approximately 12 h for 250 mg and 24 h for each of the other two higher doses. After multiple oral administration of BUDCA at day 28, peak UDCA plasma concentration occurred with a median value of approximately 3.0 to 4.0 h. Typical half-life was approximately 7 h for the 500 mg and 1000 mg doses but could not be assessed for the 250 mg dose (Figs. 3 and 4). The time of the last quantifiable plasma concentration was 24 h across doses.

Concentration-time data indicate that BUDCA is characterized by fairly rapid absorption from the gastrointestinal tract, with the majority of individuals exhibiting quantifiable BBR and UDCA plasma concentrations by the 2-h timepoint. Notably, inter-individual variability in plasma concentrations was relatively high, with coefficient of variability values of up to 128 and 181% after single dose administration for BBR and UDCA, respectively.

In general, BUDCA was well tolerated and the frequency of adverse events was similar with placebo (66%) and active drug (67%), the most frequent complaint in both groups being headache (Table 5). Three subjects withdrew from the study prior to its completion. Only one subject experienced a serious adverse event of hospitalization related to cholecystitis and cholecystectomy. This subject developed diarrhea after 14 days of treatment with BUDCA 1000 mg twice a day and study drug was stopped at that time. The subject had clinical features of acute cholecystitis and underwent cholecystectomy 6 days after stopping study drug. Interestingly serum aminotransferases were mildly elevated prior to therapy but increased to more than 10-fold above baseline values, peaking 7 days after stopping study drug. This event was judged to be possibly related to study drug by the principal investigator, but after further evaluation including a liver biopsy and case review by an external expert, was thought not to be related to study drug.

**Table 5:** Treatment-Emergent Adverse Events reported by Two or More Subjects

Adverse Event	Placebo	BUDCA Dose		
		500 mg/day	1000 mg/day	2000 mg/day
Number of Subjects	12	12	12	14
Headache	5	5	4	3
Dizziness	0	2	0	0
Nausea	1	3	1	0
Flatulence	2	0	0	0
Anorexia	0	0	3	1

## Discussion

BUDCA is a new molecular entity, made as a salt of two known therapeutic agents [21]. The present study shows that BUDCA, when administered for up to 28 days, is safe and well tolerated in a group of subjects with a history of hypercholesterolemia. Treatment-emergent adverse events were mostly minor and occurred at about the same rate in subjects treated with BUDCA as with placebo. One subject experienced a liver-related serious adverse event that was not related to study drug.

Because BUDCA is a new molecular entity, we were interested in studying its pharmacokinetic and pharmacodynamic effects. As a marker of its pharmacodynamic effect, the highest dose of BUDCA used in this study (2000 mg per day) was associated with a statistically significant decrease in serum levels of total cholesterol, LDL and non-HDL cholesterol after 14 days, sustained through 28 days of treatment. Few of the enrolled subjects had diabetes or elevated serum aminotransferases, so this study was not able to assess changes in markers of these diseases.

The BUDCA salt, when ingested, is presumed to dissociate in the gastrointestinal tract into berberine and ursodeoxycholate. This presumption was confirmed by finding differential absorption and distribution of BBR and UDCA, even though BUDCA is comprised of equimolar amounts of BBR and UDCA. Peak serum levels of UDCA were approximately 1000-fold higher than those of BBR and UDCA was detectable in the circulation an average of 2 h earlier than BBR. Interestingly, unpublished animal studies show that the concentration of BBR is approximately 10-fold higher in the liver than in serum (data on file). Detailed studies of absorption, distribution and metabolism of BUDCA in animals are underway. Human absorption, metabolism, and excretion studies are also planned.

While the mechanisms by which BUDCA exert its lipid-lowering effect remain to be elucidated, the fact that each of its two parent molecules has lipid lowering effects may have contributed to the lipid-lowering effect of BUDCA in an additive and/or synergistic manner. UDCA is a secondary bile acid, found circulating at low levels in humans although it is the predominant bile acid in some other mammalian species [16]. UDCA is used in PBC because of its choleretic benefit, its replacement of toxic bile acids in the bile acid pool and its anti-inflammatory effects. However, in this context, UDCA is thought to work by decreasing hepatic production of cholesterol while BBR appears to decrease serum cholesterol levels through increased expression of the LDL receptor via a post-transcriptional mechanism resulting in increased clearance of LDL [14, 15].

An important aspect of this trial was to study the pharmacokinetics of BUDCA so as to assist with finding a dose suitable for further study. The findings were that T_max_ (the number of hours until maximal serum levels were detected) occurred approximately 4 h (for BBR) or 2 to 4 h (for UDCA) after both single and multiple administrations of UDCA. For BBR, the typical half-life in serum ranged from approximately 8 to 10 h and was comparable across doses. For UDCA, typical half-life was approximately 7 h. After repeat dosing with BUDCA, accumulation was approximately 4-fold for BBR and approximately 2-fold for UDCA. Finally, exposure for BBR and UDCA appeared to be dose-proportional at steady state over the 4-fold range of doses evaluated in the present study. These findings support twice daily oral administration of BUDCA. Although some drug accumulation does occur after 28 days of administration, this is not excessive and indeed should enhance the actions of BUDCA, thus supporting prolonged administration.

The relatively small cohort of subjects enrolled in this study allowed accurate measurement of PK and PD (change in lipids), however the study enrolled very few patients with diabetes (only three) or hypertension (only 10). Liver enzymes were elevated in only four subjects, so the study was a not able to assess any direct benefits of these co-morbid conditions and the effects of BUDCA on plasma lipid variables in these conditions could not be assessed. Further studies will be required in these disease populations.

### Study strengths and limitations

This study has several strengths. It is the first demonstration of the pharmacokinetic profile of a new molecular entity and shows that, although BUDCA is a single entity (a salt formed from equimolar concentrations BBR and UDCA), these two metabolically active substances are handled differentially. We found that circulating levels of UDCA are about 1000 fold higher than BBR. This rather simplistic non-compartmental modelling does not take into account the local actions of BBR in the gut, the possibility that tissue concentrations may be higher than those in serum and the entero-hepatic circulation of UDCA, like other bile acids.

The study did not include a comparison with BBR alone and UDCA alone. The PK profile of UDCA is well known [22] and good quality, pharmaceutical grade berberine is not readily available to be used as a comparator. Furthermore, there is strong evidence that the actions of BUDCA amount to more than those that might be expected by the simple ingestion of each of these two components separately.

The study was a relatively small one with 12 to 14 subjects in each dose group – enough to measure PK and to assess safety and tolerability for up to 28 days, but not really enough to reliably measure pharmaceutical benefit or to compare pharmacodynamic effects in subgroups, such as males and females. Furthermore, although all subjects had to have a history of hyperlipidemia with serum LDL-c > 2.59 mmol/L, study participants generally did not have severe hyperlipidemia, making it more challenging to measure reductions in serum lipid levels.

Some of the published effects of BBR and UDCA when given separately include improvements in diabetes and hepatoprotective effects respectively. This study included very few subjects with diabetes or elevated liver enzymes due to possible fatty liver disease, so these effects could not be measured.

## Conclusion

In conclusion, BUDCA represents a novel agent consisting of a new molecular entity created from two existing agents each of which has a history of beneficial effects in liver disease, diabetes and hyperlipidemia. It has potential for use to treat hypercholesterolemia in individuals who cannot take statins, possibly as an adjunct to other agents such as ezetimibe or bempedoic acid [23]. Given experience with BBR in management of diabetes, BUDCA could potentially also be used to treat hypercholesterolemia in patients with diabetes. It is well tolerated and can be administered twice a day. Although further dose finding studies may be required, a dose of 2000 mg per day was shown to have pharmacological effect and allows this agent to undergo further testing.

## Data Availability

The authors agree to the terms of the BioMed Central Copyright and License Agreement.

## Abbreviations

NAFLD: Non-alcoholic fatty liver disease
BBR: Berberine
UDCA: Ursodeoxycholic acid
BUDCA: Berberine ursodeoxycholate
LDL-c: Low density lipoprotein cholesterol
PK: Pharmacokinetic
PD: Pharmacodynamic
